# An auditable and source-verified framework for clinical AI decision support: integrating retrieval-augmented generation with data provenance

**DOI:** 10.3389/frai.2026.1737532

**Published:** 2026-02-04

**Authors:** Fidelis Fidelis Alu, Sunkanmi Oluwadare

**Affiliations:** School of Information Technology, University of Cincinnati, Cincinnati, OH, United States

**Keywords:** artificial intelligence (AI), auditability, clinical decision support (CDS), data provenance, explainability, healthcare informatics, source verification, trustworthy AI

## Abstract

Artificial intelligence (AI) has shown promise in supporting clinical decision making, yet adoption in healthcare remains limited by concerns regarding transparency, verifiability, and accountability of AI-generated recommendations. In particular, generative and data-driven CDS systems often provide outputs without clearly exposing the evidentiary basis or reasoning process underlying their conclusions. This article presents a conceptual framework for auditable and source-verified AI-based clinical decision support, grounded in principles from evidence-based medicine, data provenance, and trustworthy AI. The proposed architecture integrates a curated medical knowledge base with explicit provenance metadata, a retrieval-augmented reasoning (RAG) engine that links generated recommendations to identifiable clinical guidelines and peer-reviewed sources, and a tamper-evident audit logging mechanism that records system inputs, retrieved evidence, and inference steps for retrospective review. This work does not introduce a new algorithm nor report a prototype implementation; rather, it synthesizes existing technical approaches into a coherent system design intended to improve traceability, clinician trust, and regulatory readiness. Key feasibility challenges are discussed, including knowledge-base governance and updating, citation fidelity in RAG architectures, bias propagation from underlying evidence, latency and usability trade-offs, privacy considerations, and alignment with emerging regulatory frameworks such as FDA Software as a Medical Device guidance and the European Union Artificial Intelligence Act. The article concludes by outlining a staged evaluation roadmap involving simulation studies and clinician-centered user research to guide future implementation and empirical validation.

## 1 Introduction

Advances in machine learning (ML) and large language models (LLMs) have raised hopes for AI-powered clinical decision support (CDS). These systems show promise in increasing diagnostic accuracy, customizing treatment recommendations to individual patients, and helping clinicians quickly find relevant medical information in fast-paced clinical settings (Workum et al., 2025). Recent research indicates that advanced LLMs can sometimes effectively match medical experts in performing tasks like case diagnosis (Workum et al., 2025). Healthcare organizations have begun piloting generative AI (GenAI) to draft clinical notes and answer clinicians’ questions, reflecting a growing interest in integrating AI assistants into clinical workflows. In a recent survey on GenAI and medical references, one in 10 doctors already use ChatGPT or similar models in their day-to-day work (Wu et al., 2024). High-profile cases, such as instances where ChatGPT correctly identified a rare condition after numerous doctors failed, have fueled optimism about the potential of AI to aid in medical diagnosis (Wu et al., 2024).

However, even with the significant promise real-world adoption has been slow because clinicians are wary of opaque algorithms that they cannot verify (Tun et al., 2025). Traditional AI often generates recommendations without explanation, leaving users unable to validate the reasoning (Amann et al., 2022). This opacity can erode trust; indeed, leading studies have repeatedly identified that lack of transparency is a major barrier to AI-CDS adoption (Tun et al., 2025; Amann et al., 2022). In practice, clinicians say they prefer recommendations that explicitly cite medical guidelines or high-quality studies (Hurt et al., 2025). This concept corresponds with the theory of evidence-based medicine (EBM) established in the 1990s, which asserts that therapeutic judgments must amalgamate personal competence with the most reliable external evidence Sackett et al. (1996). Initial informatics initiatives in Clinical Decision Support (CDS) reflected this notion: Sim et al. (2001) advocated for the incorporation of both literature-derived and practice-derived data into machine-interpretable knowledge bases to facilitate decision-making (Sim et al., 2001). Likewise, emerging guidelines from experts and regulators emphasize that AI tools should be safe, validated, and continuously monitored (Labkoff et al., 2024). In particular, regulators are increasingly focusing on transparency and traceability. For example, the U.S. FDA’s Good Machine Learning Practice principles for medical devices highlight that appropriate information about an AI system’s logic (how outputs are reached) and performance should be clearly communicated to users (U.S. Food and Drug Administration, 2024). Effective transparency ensures that information impacting patient risks and outcomes is conveyed in a way that fits the clinical user’s needs (U.S. Food and Drug Administration, 2024). Similarly, the European Union’s AI Act (2024) will classify most clinical AI systems as “high risk,” meaning they must meet rigorous requirements for risk management, transparency, data quality, non-discrimination, technical documentation, and human oversight. In medicine, where decisions affect patient lives, any AI recommendation must be reliable and explainable. One way to build trust is to make every AI-generated suggestion *source-verified*: each claim should trace back to a trustworthy medical reference. Another is to make the system fully *auditable*: every step from data input to final answer should be logged so clinicians can later review why and how a decision was made.

To address these trust requirements, we present a conceptual framework and proposal for AI-based CDS that enforces two trust-enabling features: source verification (every claim in the output traces to an authoritative source) and auditability (all inputs, sources, and reasoning steps are logged). This paper describes a theoretical design rather than an implemented system. In our design, a user’s patient query triggers a search of curated medical literature and databases; an AI module then synthesizes an answer that includes exact citations to the retrieved sources. Meanwhile, a secure audit trail records which data and references were used and how the final suggestion was derived. This way, clinicians can see *why* a recommendation was made and trace it back to original evidence, rather than accepting an unexplained suggestion. This design allows clinicians to both verify recommendations in real time and review them retrospectively, providing transparency and accountability.

Our contributions are as follows: (1) we define design principles for auditable, source-verified AI in healthcare, aligned with established trust factors such as transparency, reliability, and human-centered design (Tun et al., 2025; Amann et al., 2022); (2) we propose a conceptual architecture for AI-CDSS that integrates evidence retrieval, citation, and audit logging as a synthesis of existing concepts rather than a built prototype; and (3) we situate this framework within the context of regulatory expectations and provenance research, for example, aligning with FDA’s Software as a Medical Device guidance and the EU AI Act’s transparency and oversight requirements outlining a roadmap for future implementation and evaluation through simulation studies and clinical user trials.

The following sections review related work on AI trust and provenance, describe the proposed framework in detail, and discuss implications, limitations, and future steps.

## 2 Background and related work

The pursuit of source verification in clinical decision support (CDS) is not new; it has been a foundational principle of Evidence-Based Medicine (EBM) since the 1990s (Sackett et al., 1996). Early medical informatics initiatives focused on constructing machine-interpretable knowledge bases encoded as static, rule-based systems (Sim et al., 2001). Because these systems used deterministic logic derived directly from curated guidelines, they were intrinsically auditable: the chain from input to recommendation could be reconstructed, and the underlying rules could be manually inspected.

The current generation of LLM based CDS systems presents a qualitatively different paradigm. Rather than applying fixed rules, LLMs engage in probabilistic reasoning over high-dimensional representations. This flexibility enables richer clinical dialogue but also introduces well-documented risks, including hallucination and unstable citation behavior. Traditional EBM-style CDS architectures were never designed to manage these risks. The novelty of the present framework lies in explicitly anchoring probabilistic model behavior to a deterministic evidence base, and in treating verifiable provenance not as a user-interface feature but as a core architectural constraint.

Several systems already incorporate retrieval mechanisms or citation displays alongside generated answers. These RAG systems surface evidence-based documents with AI-generated summaries. However, these approaches typically operate at the level of “citation-assisted generation,” in which evidence is attached to the output after the reasoning has already occurred. In contrast, the framework proposed here embeds verification into the inference chain itself: the model is constrained to reason only over verified retrieved sources, and the full retrieval and decision pathway is recorded in an auditable log. Thus, the contribution of this work is not merely adding citations, but converting provenance into a first-class control mechanism for clinical AI behavior.

Trust in CDS systems has received increasing attention in recent years. Recent surveys and reviews identify core factors affecting clinician trust: transparency (how the system’s output is generated), usability, clinical reliability, validation, ethics, and human-centered design. In one systematic review of 27 studies, Tun et al., (2025) summarize eight themes crucial to trust in AI-CDSS: (1) system transparency, (2) user training, (3) usability and workflow integration, (4) clinical reliability and consistency, (5) validation across contexts, (6) ethical and fairness concerns, (7) patient-centeredness, and (8) customization and clinician control (Tun et al., 2025). Common barriers include algorithmic opacity and lack of user training, whereas enabling factors include transparent design and proven reliability. In particular, system transparency, often tied to explainability, repeatedly comes up as essential for trust (Ferrario and Loi, 2022). Consistent with this, many authors argue that AI recommendations should come with explanations or source links so clinicians can verify them. For example, Hurt et al. (2025) evaluated the OpenEvidence AI platform, which provides answers explicitly citing evidence-based medicine sources; physicians rated its recommendations as very clear, relevant, and well-supported by citations. In feedback, users expressed that seeing official guideline references boosted their confidence, helping prevent “AI hallucinations” (false information) by anchoring each claim to real documents. These findings suggest that integrating evidence retrieval can make AI-CDSS outputs more trustworthy (Hurt et al., 2025).

However, as Amann et al. (2022) point out, transparency is not one-dimensional: “Explainability is but one measure of achieving transparency; other measures include detailed documentation of datasets and algorithms, as well as open communication of the system’s capabilities and limitations” (Amann et al., 2022). In other words, even if a system does not produce a human-readable explanation, it can still be trustworthy if it documents its workings and provides evidentiary support.

Regulatory and professional guidelines echo these points. Recent mandates, such as the European Union (2024) and the U.S. Food and Drug Administration guidelines for transparency in AI-enabled medical devices (2024). Labkoff et al. (2024) also convened an expert panel which issued recommendations for AI-enabled CDS. Key among them are building *safe and trustworthy* systems and establishing robust *validation, verification, and certification* processes. The panel emphasized national-level monitoring (e.g., registries or mandatory reporting for AI tools) and thorough documentation and training for end users (Labkoff et al., 2024). Their envisioned framework covers model training, explainability, continuous monitoring, and regulatory oversight—essentially a lifecycle approach to AI safety (Labkoff et al., 2024). Such measures align with our goals: by design we enforce both transparency (through source links and documentation) and the capacity for safety monitoring (through audit logs that regulators or hospitals could review).

**Figure 1:**
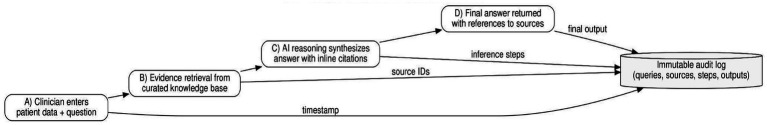
Illustrates the workflow: **(A)** Clinician enters patient data and question; **(B)** system retrieves relevant evidence from its curated database; **(C)** reasoning engine synthesizes an answer, marking each point with reference IDs; **(D)** final answer and references are presented to the user, while a background module logs all steps.

Other researchers have explored related ideas. Explainable AI (XAI) has been widely studied in clinical contexts, but simply providing *post-hoc* explanations can be misleading or insufficient. A different line of work focuses on provenance and evidence retrieval. For example, OpenEvidence is an AI system that answers doctors’ questions by drawing on “trusted sources” in evidence-based medicine (Hurt et al., 2025). In a recent evaluation, it returned clear, relevant recommendations (with citations) for primary care cases, reinforcing physician decisions (Hurt et al., 2025). This shows the promise of *source-driven AI*: clinicians value seeing concrete references. Our approach builds on this concept but embeds it into a full CDS platform with integrated logging for accountability (Figure 1).

Finally, data provenance tools and standards suggest technical ways to implement auditability. A recent study defines data provenance as “attributes about the origin of health information at the time it is first created and tracks the uses and permutations of the health information over its lifecycle.” It is important to note that outside of healthcare, the importance of provenance and audit logs for accountability is long recognized in computer science and data engineering. Provenance systems often rely on detailed logs, cryptographic checks, or even blockchain techniques to ensure data integrity and traceability (Ahmed et al., 2023). These mechanisms provide accountability: every read or write action can be traced, enabling audits in case of errors or misconduct (Ahmed et al., 2023). In particular, tamper-evident audit logs can offer a comprehensive record of system events, enabling *traceability* and *accountability*: any read/write or inference step can be examined after the fact. By logging each step of data collection and processing, provenance tools allow investigators to trace errors or biases back to specific inputs. More generally, audit logs (also called audit trails) catalog every system event (data read/writes, inferences, user actions) with timestamps and user IDs. Well-designed audit logs offer “transparency and integrity” of records, serving as an evidential support layer (Regueiro et al., 2021).

To prevent tampering, modern approaches often use cryptographic or distributed ledgers. For example, blockchain-based audit mechanisms have been proposed for healthcare records and AI systems. Regueiro et al. (2021) describe a blockchain audit-trail system where each log entry is stored on a distributed ledger, so it “cannot be modified at all” once written. This ensures immutability: once information is on the chain, it “can never be deleted” and thus any audit trail remains fully traceable In short, blockchains offer integrity and non-repudiation for audit logs (Regueiro et al., 2021). Similar ideas appear in other domains: financial institutions, digital forensics, and distributed systems routinely require tamper-evident logs (often append-only databases or ledgers) to enforce accountability. However, the computational overhead and latency of public blockchains can be a barrier in clinical settings; thus, our framework prioritizes permissioned architectures and hybrid storage models to ensure performance matches the speed of clinical care. The cryptographic provenance techniques developed in data engineering thus provide models for clinical AI: every data point, model update, and AI recommendation can be cryptographically timestamped and logged so that any downstream user (clinician, auditor or regulator) can retroactively verify exactly what happened.

In summary, past work on XAI, trust surveys, regulatory frameworks, and data provenance all point to the same conclusion: to be accepted in medicine, AI systems must be transparent in evidence and fully auditable in operation. We next describe our proposed framework that puts these principles into practice.

## 3 Methods (framework design)

**Figure 2:**
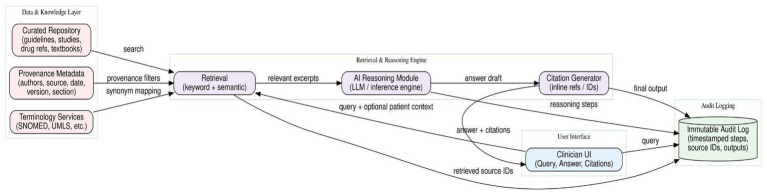
Shows a block diagram with modules and data flow: user interface, knowledge base, retrieval engine, AI model, citation generator, and audit logger. The diagram shows the major components and data flow. The clinician interacts via a user interface which sends the query (with relevant patient data) to the system. The nowledge ase (curated medical repository) is queried by the etrieval odule, which returns relevant documents. The AI easoning ngine (LLM) then generates an answer using those documents, and a itation enerator ensures references are attached to the answer. Meanwhile, an udit ogger records each step (query, retrieved sources, generated answer, etc.) into a secure log storage. Finally, the answer with its citations is displayed to the clinician. The numbered sequence (1–4) indicates the workflow: (1) ser’s query in, (2) evidence retrieved, (3) answer produced with citations, (4) answer and references returned to user, with all steps logged in the background.

The framework was developed by synthesizing insights from the above literature. We identified core requirements for trust (transparency, reliability, user control) and translated them into system design requirements. In brief, we require an AI-CDSS where: (1) every recommendation is backed by identifiable sources; (2) every stage of processing is recorded; and (3) outputs are delivered in a user-friendly manner. To meet these goals, we sketched a high-level architecture with three principal components: a Data/Knowledge Layer for authoritative content, a Retrieval and Reasoning Engine to answer queries with evidence, and a User Interface with Audit Logging (Figure 2 presents the high-level architecture). Each design decision is grounded in prior work: for example, our evidence retrieval mechanism is inspired by platforms like OpenEvidence (Hurt et al., 2025), and our audit log design follows best practices from provenance research (Ahmed et al., 2023). We then iteratively refined this design to ensure it could theoretically satisfy the trust themes identified by Tun et al., (2025) and the recommendations of Labkoff et al. (2024). No empirical experiments or data collection were involved, as this is a conceptual, future-oriented system design.

### 3.1 Proposed framework

The system architecture (see Figure 2) consists of the following major modules:

#### 3.1.1 Data and knowledge layer

This layer serves as the framework’s foundational repository, curated to ensure both clinical validity and technical traceability. It integrates structured data (e.g., clinical guidelines, drug interaction databases) with unstructured sources (e.g., PubMed abstracts and peer-reviewed medical textbooks).

Provenance and evidence grading: Every content item is tagged with comprehensive provenance metadata, including authors, publication dates, and source types. To address the requirement for high-quality evidence, we also incorporate evidence grading [e.g., GRADE levels (Guyatt et al., 2008)] into the metadata. This allows the system to record not just where a fact came from, but the clinical strength of that evidence. For example, if the system utilizes a specific fluid resuscitation guideline, it records the exact section and version, ensuring that the “source of truth” remains unambiguous during a retrospective audit.

Terminology services: This layer houses the standardized ontologies, specifically SNOMED-CT (Donnelly, 2006) and UMLS (Bodenreider, 2004), that the system uses to bridge the gap between messy clinical language and formal medical evidence. By maintaining these terminology services at the data layer, the system can map heterogeneous user queries (e.g., “high blood pressure”) to unified concepts (e.g., “Hypertension”), ensuring that retrieval is based on semantic meaning rather than just keyword matching.

Bias mitigation and curation: To address the potential for AI to amplify medical disparities, this layer includes a Bias Auditing Protocol. During the curation process, sources are screened for representative diversity in their study populations. If a guideline or study is identified as having significant demographic gaps, it is flagged within the metadata. This allows the Reasoning Engine to either weigh that evidence differently or provide an explicit “Bias Warning” to the clinician, ensuring that “source-verification” also encompasses an assessment of evidence equity.

Maintenance and integrity: The knowledge base is not static; it is updated continuously as new evidence emerges. Critically, these update operations are themselves recorded as transactions in the audit log. This creates a history of “knowledge evolution,” allowing an auditor to see exactly what the “state of medical knowledge” was at the time any specific recommendation was made. This ensures the system remains current while maintaining a clear, identifiable provenance trail for every fact in the repository.

#### 3.1.2 Retrieval and reasoning engine

When a clinician poses a patient-specific question (e.g., “What is the next best step for a diabetic patient with high cholesterol?”), the system first retrieves relevant evidence. Initiates a multi-stage processing pipeline. To address the inherent ambiguity of clinical language, the system first passes the query through a Semantic Mapping and Disambiguation sub-layer. Rather than relying on simple keyword matching, which fails to account for synonyms (e.g., “SOB” vs. “Shortness of Breath”) or polysemy (e.g., “MS” meaning Multiple Sclerosis or Mitral Stenosis), the engine utilizes terminology services integrated with SNOMED-CT (Donnelly, 2006) and UMLS (Bodenreider, 2004). This layer performs “entity linking,” mapping unstructured user input and patient record data into normalized medical concepts. This ensures the system understands the clinical intent before it ever queries the knowledge base.

Following disambiguation, the search module matches keywords in the query (including data from the patient’s record) against the knowledge base and fetches all pertinent sources (for instance, the latest diabetes and lipid management guidelines, and recent clinical trials on cholesterol-lowering therapy). These documents serve as the factual foundation. Next, an AI reasoning module (built on a RAG architecture) processes the retrieved texts together with the patient data to generate a recommendation. Crucially, the output is formulated to link each claim back to specific sources: the text includes inline citations or reference IDs corresponding to the original documents. This is analogous to how a clinician might cite a guideline when explaining a decision. By construction, every assertion in the recommendation is supported by a known source from the curated repository. In practice, the answer might appear as plain-language guidance with superscript numbers or footnotes pointing to referenced studies. This approach mirrors evidence-based practice and was shown effective in prior work: for example, Hurt et al. (2025) found that providing clear citations in AI-generated answers made them highly credible to physicians (Hurt et al., 2025). Because all sources are recorded, the chain of reasoning can be revisited later if needed. However, a significant technical challenge persists: RAG systems can sometimes misattribute citations or fail to retrieve the most relevant context. Our framework incorporates specific mitigation strategies:

1. Confidence scoring: Each retrieved passage is assigned a relevance score; only those above a high-certainty threshold are used in reasoning.

2. Ensemble retrieval: Using multiple search algorithms (e.g., semantic search plus BM25) to ensure the most relevant context is captured.

3. *Post-hoc* verification: A secondary “critic” agent verifies that the final generated recommendation is actually supported by the cited text before it is displayed to the clinician.

#### 3.1.3 Audit logging Interface

The system maintains an immutable audit log capturing the full decision process. At runtime, every action is recorded with a timestamp. The log includes the original user query, any patient data elements accessed (stored as cryptographic hashes to protect privacy), the unique identifiers (IDs) of retrieved sources, and the exact steps the reasoning engine took. Concretely, the inference_chain field would capture the specific retrieved sentences or data points used to support each claim in the final output, along with any intermediate reasoning steps.

In technical terms, the system writes structured log entries (akin to database transaction logs) for each operation. A typical log entry would contain fields such as:

{timestamp, user_id, session_id, query, patient_data_accessed (hashed), retrieved_doc_IDs, inference_chain, final_output_ID}.

To address concerns regarding computational overhead and clinical latency, this framework proposes a hybrid storage model utilizing a permissioned distributed ledger (e.g., Hyperledger Fabric) (Androulaki et al., 2018). Unlike public blockchains, a permissioned DLT ensures that only authorized institutional nodes participate, providing sub-second latency required for time-sensitive clinical settings. In this model, the “heavy” clinical data remains in secure, HIPAA-compliant off-chain storage, while only the cryptographic “hash” (the digital fingerprint) of the log entry is committed to the ledger.

This architecture makes the log tamper-evident and facilitates efficient querying during audits. Each log entry uses unique identifiers so that an answer (e.g., “Answer #1234”) can be linked to its constituent sources (“used Guideline A, sec 5.3; Trial B, NEJM 2018,” etc.). Access controls and digital signatures ensure that if a clinician or regulator later questions a recommendation, they can “replay” the audit record. They would see exactly what information the AI saw and how it combined those facts, satisfying the transparency requirements of the European Union (2024) and the U.S. Food and Drug Administration (2024) guidelines As Ahmed et al. (2023) note, logs of this kind provide a “comprehensive record” that enables traceability, accountability, and auditing of the system. In our design, the log operates invisibly in the background: users receive only the final answer and its citations, but the full provenance is preserved behind the scenes for review if necessary.

By combining these components, the framework achieves the stated goals. Source verification is ensured because every output includes explicit references to the original literature. Auditability is ensured because the entire chain of data and reasoning is recorded. The user interface is kept straightforward: clinicians see clear recommendations in everyday language, numbered by reference (not raw model outputs or code). In summary, the system is designed so that users can trust an AI answer in the same way they trust clinical guidelines, by checking the cited evidence and, if necessary, reviewing the logged reasoning.

The above three modules work in concert to fulfill our key objectives. The output to the clinician is a concise, plain-language recommendation (e.g., a diagnostic or treatment suggestion) with clearly marked references supporting each part of it. The clinician can click on a reference to see, for example, the guideline excerpt or PubMed abstract that was the source. In this way, source verification is achieved at the point of care. Simultaneously, auditability is achieved behind the scenes by logging everything. If questions arise later (e.g., “Why did the AI recommend this? On what basis?”), the log can be consulted to reconstruct the scenario. The user interface remains as straightforward as possible; it might look like a normal CDS advisory or an entry in a clinical note with superscript citations. We intentionally do not expose raw model internals or complicated probability scores to the end-user, as that could confuse or overwhelm clinicians. Instead, clinicians see advice that resembles an annotated guideline or an evidence-based summary. This respects the fact that usability and workflow integration are critical for adoption. The design lets physicians focus on the content of the recommendation, with the option to drill down into sources if they want more detail.

In theory, this architecture addresses multiple trust themes. It provides transparency (via explicit citation of sources), reliability (by grounding outputs in vetted, up-to-date evidence), and accountability (through the audit log of decision-making). These theoretical strengths, of course, must be proven in practice. We stress again that this work is *conceptual*. We have not built or tested this system yet, so its practical effectiveness is unconfirmed. The framework assumes the existence of a comprehensive, well-maintained knowledge base and an AI model that can integrate with it, both of which pose non-trivial challenges. In the next sections, we discuss these feasibility issues and outline how we plan to evaluate the approach.

#### 3.1.4 Integration and interoperability

A practical deployment of the proposed framework requires integration with existing clinical information systems, particularly electronic health record (EHR) platforms. In real-world environments, EHR interoperability remains a significant barrier to clinical decision support adoption, and any trustworthy AI architecture must therefore explicitly account for these constraints. Rather than assuming frictionless integration, our framework is designed to align with established interoperability standards such as HL7 v2, HL7 FHIR, and SMART-on-FHIR.

In an implemented system, FHIR resources (e.g., Patient, Observation, Condition, MedicationRequest) would be used to ingest structured patient context data into the reasoning pipeline. However, variation in vendor-specific FHIR implementations, incomplete data fields, and site-specific customization pose substantive challenges. Additionally, a substantial proportion of clinically relevant information exists in unstructured clinical notes, which requires entity extraction and disambiguation prior to use. Mapping between terminologies such as SNOMED CT, ICD-10, RxNorm, and UMLS is similarly non-trivial and introduces opportunities for semantic drift.

Accordingly, interoperability in this framework is not treated as an engineering inevitability but as a design constraint. Future prototype development will include incremental integration layers tested in controlled pilot environments rather than assuming universal deployment across heterogeneous EHR infrastructures.

#### 3.1.5 Human-centered design: mitigating cognitive load

Clinicians operate under significant time pressure, and any decision support tool that increases cognitive load risks non-adoption regardless of technical sophistication. While our framework emphasizes explicit source citation and auditability, presenting full evidentiary chains by default could overwhelm users rather than assist them. To address this, the envisioned implementation adopts a design principle of progressive disclosure of information.

In practice, this means that the system would initially present concise, high-level recommendations accompanied by a small number of primary citations with the highest evidence strength. Users could then expand additional layers such as detailed rationale, secondary sources, evidence synthesis, and full audit trail only when desired. This approach balances transparency with efficiency by surfacing depth only when needed. In addition, user interface mechanisms such as collapsible sections, summarization of long passages, warning banners for low-confidence recommendations, and optional “audit view” modes are intended to support rapid clinical decision making without sacrificing traceability.

By explicitly prioritizing workflow alignment and cognitive load reduction, the framework seeks not only to be technically trustworthy but practically usable in real clinical environments.

### 3.2 Planned evaluation

Our goal with this evaluation plan is to show that this framework is not just a concept, but something that can actually work in practice. We focus on three things: whether the system technically does what we claim, whether clinicians can realistically use it, and whether it improves decision-making in a measurable way. We are not claiming full live deployment yet; instead, we are building strong feasibility evidence step-by-step.

#### 3.2.1 Phase 1: Simulation testing

First, we will test the system in-silico using PubMedQA (Jin et al., 2019) and MedQA (Jin et al., 2020), along with clinical vignettes. Here we will look at accuracy of answers, but more importantly, how well citations actually support the statements the model makes. We will measure citation precision and recall, hallucinated citations, consistency across repeated runs, and the extra time added by verification and logging. We will also run the system with and without the verification layer so we can clearly see what the verification component is adding.

#### 3.2.2 Phase 2: Clinician study with sample size and power

Next, we will study how clinicians actually use the system. We plan to recruit about 50–60 clinicians (a mix of residents and attendings). Reviewer 3 asked specifically about power, so we address that directly here: based on effect sizes reported in earlier work on AI-CDSS trust and workload measures, a sample of this size should give us around 80% power to detect meaningful differences in trust and cognitive workload at *α* = 0.05. In this study, clinicians will work through realistic cases using (a) no AI support, (b) a normal RAG tool with citations, and (c) our framework. We will measure time-to-decision, usability (SUS), cognitive load (NASA-TLX), and trust in automation. We will also actually ask them how it felt to use it—did transparency help, or did it slow them down?

#### 3.2.3 Phase 3: Decision quality and outcome proxies

Finally, we will look at whether the system improves decision quality. We are realistic here: full patient-outcome trials are beyond the scope of this paper. So instead we will use reasonable proxies, such as guideline-concordant decisions, avoiding contraindicated medications, correct dosing, correct screening intervals, and when clinicians choose to override the AI. These are practical indicators that link directly to patient safety without pretending we are already running a live trial.

### 3.3 Clinical simulation

To demonstrate the feasibility of the proposed framework, we provide a functional walkthrough using a high-stakes clinical scenario: the management of a patient with suspected sepsis in an Emergency Department. While this is a conceptual analysis, the simulation illustrates how the disparate layers of the architecture, Semantic Mapping, Retrieval-Augmented Generation (RAG), and Distributed Ledger Technology (DLT), function as a unified system to ensure safety and accountability.

#### 3.3.1 Step 1: Input and semantic disambiguation

The process begins when a clinician enters a bedside query: *“The patient has suspected sepsis and low blood pressure. What is the recommended fluid protocol?”* Before retrieval occurs, the engine identifies the patient’s vitals from the integrated Electronic Health Record (EHR), noting a mean arterial pressure (MAP) of 60 mmHg.

To resolve the inherent linguistic ambiguity in clinical language, the Semantic Mapping Layer maps the clinician’s term “low blood pressure” to the normalized concept “Hypotension” (SNOMED-CT: 45007003) (Donnelly, 2006). This step ensures that the subsequent retrieval is based on standardized medical terminology rather than a simple keyword match, grounding the AI’s “understanding” in recognized clinical ontologies (Bodenreider, 2004).

#### 3.3.2 Step 2: Source-verified retrieval (RAG)

The Retrieval Layer queries the curated Knowledge Base, fetching the *Surviving Sepsis Campaign (SSC) International Guidelines* (Evans et al., 2021). Using a RAG architecture, the system extracts the specific intervention for fluid management.

Simultaneously, a Bias Mitigation protocol evaluates the metadata of the retrieved source. In this instance, it confirms the SSC guidelines are a high-grade consensus source but flags the “Low Quality of Evidence” associated with the specific fluid volume recommendation (30 mL/kg) as defined by the GRADE levels (Guyatt et al., 2008). This proactive check ensures the recommendation is presented with its necessary clinical nuances, preventing the system from treating all guidelines as having equal evidentiary weight.

#### 3.3.3 Step 3: Recommendation and user interface

The system generates a concise, actionable recommendation for the clinician:

Recommendation: Initiate 30 mL/kg IV crystalloid fluid immediately for suspected sepsis-induced hypoperfusion. Source: *Surviving Sepsis Campaign 2021, Recommendation #12 (GRADE: Strong recommendation, Low quality of evidence) [View Full Text]*

This interface design prioritizes usability and minimizes cognitive load. By presenting the recommendation first while keeping the supporting evidence accessible via a direct link, the system provides “just-in-time” guidance that respects the time constraints of acute care. This transparent linkage fulfills the requirement for human-in-the-loop oversight mandated by emerging regulatory frameworks such as the European Union (2024).

#### 3.3.4 Step 4: Forensic audit logging

As the clinician acts on the recommendation, the Audit Logging Interface executes a background transaction. To ensure the sub-second latency required in time-sensitive clinical settings, the framework utilizes a hybrid storage model on a permissioned distributed ledger (Hyperledger Fabric) (Androulaki et al., 2018).

While the “heavy” clinical data remains in the hospital’s HIPAA-compliant storage, a structured log entry, containing the original query, the specific retrieved passage from the SSC guidelines, and a cryptographic hash of the reasoning chain, is committed to the ledger. This creates an immutable, timestamped record of the exact evidence provided to the clinician. This “source-to-decision” trail provides the technical provenance necessary to satisfy FDA transparency guidelines (2024) and institutional liability requirements.

## 4 Discussion

Although no working system has been built, the proposed design illustrates how known methods could be combined to meet trust requirements for medical AI. First, by anchoring each claim in verified sources, the system would mitigate the risk of unsupported “hallucinations” associated with most LLMs. Any factual statement it makes can be directly checked against the cited guideline or study text. This is a critical safeguard: physicians participating in Hurt et al.’s (2025) study noted that seeing official citations increased their confidence in the AI’s suggestions (Hurt et al., 2025). In effect, the AI operates like a junior physician who always cites a textbook when giving advice. Second, the transparent linkage to evidence addresses a top concern from the trust literature. Tun et al., (2025) emphasize that system transparency, being able to understand how outputs are derived is essential for user trust (Tun et al., 2025). Our design provides transparency at two levels: both by exposing evidence sources to the user and by maintaining an internal log for deeper inspection.

Moreover, this framework aligns with recommendations from informatics experts. Labkoff et al. (2024) advocated a lifecycle approach where AI tools are continuously monitored and validated (Labkoff et al., 2024). A full audit log, as we describe, is exactly the kind of safety monitoring tool they envisage: it would allow healthcare organizations to track AI performance over time and investigate any adverse events. From an ethical standpoint, detailed logging and source documentation also support fairness and liability concerns by showing exactly what information influenced a decision (Ahmed et al., 2023).

Finally, the user experience is kept clinician-centered. Rather than presenting complex model internals, the system outputs resemble annotated clinical guidelines. Physicians can focus on the medical content and (if desired) review the cited references themselves. This respects the finding that *usability and workflow integration* are key enablers for AI adoption (Tun et al., 2025). In summary, the proposed architecture conceptually addresses multiple trust themes identified in recent reviews like that of Tun et al., (2025). It offers transparency through explicit citation, reliability by grounding outputs in vetted guidelines, and accountability through audit logging. These theoretical strengths must ultimately be confirmed in practice. 4.1 Limitations.

This work is purely conceptual. We have not implemented, tested, or empirically validated the proposed system. As such, its practical effectiveness is unknown. The framework assumes the existence of a comprehensive, up-to-date medical knowledge base; building and maintaining that repository (with accurate provenance metadata) would itself be a major challenge. This knowledge base curation presents a monumental task involving content licensing, continuous updates to reflect new evidence, resolution of conflicting guidelines, and mitigation of inherent biases in the source materials. The system’s reliability is entirely contingent on overcoming these challenges.

We have also assumed that a language model can reliably synthesize coherent recommendations from retrieved text; in practice, integrating GenAI with strict citation requirements may require careful engineering. As noted in Section 3.1, the inherent limitations of RAG architectures, particularly citation misattribution, pose a significant technical risk to the core source verification feature. Mitigation strategies will be essential in any future implementation.

Other limitations include performance and usability trade-offs. Retrieving and logging every step could introduce latency or complexity that might impede real-time clinical use. Users may also be overwhelmed by too many references or auditing details if the interface is not well-designed. Moreover, linking to sources does not automatically ensure correctness if the underlying evidence is outdated or biased. This “garbage in, garbage out” problem means the framework’s reliability is entirely contingent on the quality and lack of bias in the underlying knowledge base. Finally, strict logging raises privacy and security considerations; the design would need to ensure patient data are handled in compliance with regulations like HIPAA and GDPR. All these issues would need to be addressed in future implementations.

### 4.1 Future work

Key next steps include implementation and evaluation. We plan to develop a prototype system based on this framework and integrate it with electronic health records for specific use cases (e.g., managing hypertension or diabetes). User testing with clinicians will be needed to assess whether the source-verification and audit features actually improve trust and decision quality in practice. Quantitative evaluations could compare the accuracy, usability, and user confidence of our system versus a conventional AI-CDS.

Other research directions include optimizing the retrieval and reasoning components (e.g., experimenting with different retrieval algorithms and language models) and developing robust methods to mitigate RAG limitations, such as citation verification modules. Improving the audit log. We will also need to explore how to update the knowledge base continually and how to streamline the user interface so clinicians can easily navigate the references and logs without undue burden. Finally, it will be important to engage with ethicists and regulators to ensure that the framework meets legal and professional standards for AI in medicine.

### 4.2 Bias mitigation and evidence equity

A significant concern in clinical AI is that “source-verification” alone does not guarantee accuracy if the underlying evidence is fundamentally flawed. Reviewers and ethicists correctly point out that medical literature often contains inherent biases, particularly regarding demographic representation. If the knowledge base consists of studies with skewed populations, the AI’s “verified” recommendations will merely amplify those disparities, a technical “garbage in, garbage out” scenario.

To address this, our framework proposes a Diversity-Audit during the knowledge base curation phase. This is a proactive metadata layer that flags the demographic composition of the source material. The necessity for this is underscored by current statistics: for example, according to FDA 2020 drug trial snapshot while Black/African American individuals make up approximately 13% of the U.S. population, they accounted for only 8% of clinical trial participants for FDA-approved drugs in 2020. Similarly, Hispanic/Latino participation was roughly 11% despite representing 19% of the population (U.S. Food and Drug Administration, 2020).

By implementing a Diversity-Audit, the system can alert clinicians when a recommendation is based on evidence with poor representative parity. This allows the framework to move beyond simple technical verification toward a model of “Evidence Equity,” where the AI provides not just the most cited answer, but the one most relevant to the specific demographic context of the patient being treated.

## 5 Conclusion

Trustworthy AI for medicine requires careful design. We have presented a theoretical design for an AI-powered clinical decision support system that is *auditable* and *source-verified*. By linking every recommendation to explicit medical evidence and by logging all reasoning steps, this approach could help ensure that AI enhances medical decision making without compromising safety or transparency. The framework synthesizes ideas from trustworthy AI research and data provenance literature. Although yet to be implemented and tested, it suggests one path toward AI-CDSS tools that clinicians might genuinely trust: ones where every claim can be traced to its origin, and every decision can be retrospectively examined. As AI tools become more common in clinics, adopting these standards will be crucial. In the future, we envision that combining this framework with broader evaluations and continuous monitoring should help ensure that AI truly enhances, rather than undermines, medical decision making.
